# Genomic data reveal unexpected relatedness between a brown female Eastern Bluebird and her brood

**DOI:** 10.1002/ece3.10851

**Published:** 2024-01-24

**Authors:** Joseph L. Schroeder, Alexander J. Worm, Andrew D. Sweet, Virginie Rolland

**Affiliations:** ^1^ Department of Biological Sciences Arkansas State University State University Arkansas USA

**Keywords:** atypical plumage, kin selection, population bottleneck, sexual selection

## Abstract

Because plumage coloration is frequently involved in sexual selection, for both male and female mate choice, birds with aberrant plumage should have fewer mating opportunities and thus lower reproductive output. Here we report an Eastern Bluebird (*Sialia sialis*) female with a brown phenotype that raised a brood of four chicks to fledging. The brown female and her mate were only related to their social offspring to the second degree and one of the offspring was a half‐sibling. We propose four family tree scenarios and discuss their implications (e.g., extra‐pair paternity, conspecific brood parasitism). Regardless of the tree, the brown female was able to find a mate, which may have been facilitated by the bottleneck created by the severe snowstorms in February 2021.

## Graphical abstract

We report on a female bluebird (*Sialia sialis*) with a brown phenotype that successfully found a mate and raised a brood of four chicks, which goes against expectations under sexual selection. The brown female and her mate were only related to their social offspring to the second degree and one of the offspring was a half‐sibling. We explored four possible family trees and surrounding circumstances (e.g., kin selection, population bottleneck) that could have led to this observation.

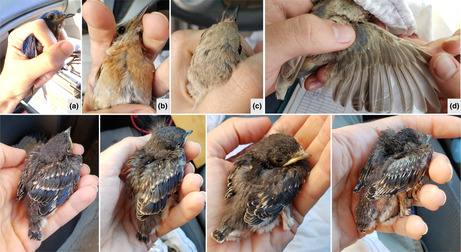

## Résumé

Parce que la coloration du plumage est fréquemment impliquée dans la sélection sexuelle, tant pour le choix du partenaire mâle que femelle, les oiseaux avec un plumage aberrant devraient avoir moins d'opportunités d'accouplement et donc un rendement de reproduction réduit. Nous rapportons ici le cas d'une femelle Merlebleu de l'Est (*Sialia sialis*) avec un phénotype brun qui a élevé une nichée de quatre poussins jusqu'à l'envol. La femelle brune et son partenaire n'étaient liés à leur progéniture sociale qu'au second degré et l'un des poussins était un demi‐frère ou une demi‐sœur. Nous proposons quatre scenarios d'arbre généalogique et discutons de leurs implications (par exemple, paternité hors couple, parasitisme conspécifique), mais quel que soit l'arbre, la femelle brune a pu trouver un partenaire, ce qui a peut‐être été facilité par le goulot d'étranglement créé par les fortes tempêtes de neige en février 2021.

## 1 INTRODUCTION

For many avian species, plumage coloration plays a vital role in sexual selection. Males have evolved ornamentation (including vivid plumage colors) due to the selective pressures of female choice. However, recent research suggests sexually selected traits are also common in females (Dale et al., 2015; Polo et al., 2021; Siefferman & Hill, 2005), especially in sexually dichromatic species with biparental care (Hernández et al., 2021). Plumage coloration includes pigmentary and structural coloration. Structural coloration (blues, greens, purples, and iridescent gradients) results from light scattering by feather nanostructures, which for non‐iridescent colors are in the spongy matrix of feather barbs (Dyck, 1971). This matrix sits between a keratin cortex and a basal layer of eumelanin granules around an air vacuole (Shawkey et al., 2005).

Numerous aberrations in plumage coloration can occur for both pigmentary and structural coloration (Guay et al., 2012; van Grouw, 2013, 2021). One such aberration, in mammals and birds, is the brown phenotype (also called the brown mutation), which can be caused by several mutations to the TYRP1 gene. These mutations result in incomplete synthesis of eumelanin (Domyan et al., 2014; Kobayashi et al., 1998). Therefore, all parts of the organism that should normally be brown and black range from pale cream to dark brown (Domyan et al., 2014; van Grouw, 2021).

Birds with the brown phenotype are common in the wild, but are frequently misidentified (e.g., as leucistic or diluted; van Grouw, 2012, 2013), probably because brown plumage bleaches over time from prolonged exposure to sunlight (van Grouw, 2021). Furthermore, because TYRP1 is on the Z chromosome in birds, females (ZW) are more likely than males (ZZ) to express the brown trait.

The frequency of atypical plumage might increase in case of population bottlenecks. A bottleneck reduces mating opportunities, which might increase the chance that a bird with atypical plumage successfully breeds (Bensch et al., 2000). Population bottlenecks can also cause inbreeding, reduced genetic diversity, and increased frequency of deleterious recessive alleles (Kirkpatrick & Jarne, 2000; Luikart et al., 1998; Nei et al., 1975). Notably, the effects of a genetic bottleneck can be pronounced for Z‐linked loci (such as the TYRP1 gene) due to the smaller effective population size of Z chromosomes (Pool & Nielsen, 2007, Schield et al., 2021). Thus, the frequency of birds with atypical plumage may be more frequent after a bottleneck (Bensch et al., 2000).

Among bluebirds (*Sialia* spp.), reports of individuals with atypical plumage have been anecdotal and mostly at the chick stage (Ellis & Parish, 1988; Hanebrink, 1985; Peak, 2022), with no monitoring of their survivorship or future ability to reproduce successfully. Eastern Bluebirds (*S. sialis*) are sexually dichromatic with males more ornamented than females. However, both sexes have structural blue plumage on their head, back, and wings; a white belly; and a chestnut‐colored breast due to a combination of eumelanin and phaeomelanin pigments (Siefferman & Hill, 2003). In this species, both pigmentary and structural coloration are involved in sexual selection for both sexes and reproductive success is higher for both males and females with more−ornamented structural plumage (Grindstaff et al., 2012; Siefferman & Hill, 2003, 2005). Because the breast chestnut color is the result of mostly phaeomelanin pigments, a brown individual would retain its chestnut breast but would have a cream‐beige plumage on head, back, and wings.

Two severe snowstorms swept the United States in February 2021, causing mass mortality in birds (Flesher & Stengle, 2021), including Eastern Bluebirds (unpublished data). A year later, we discovered an adult female Eastern Bluebird with the brown phenotype in Jonesboro, Arkansas, USA. We monitored her nest of four eggs for about 3 weeks. With her partner, which exhibited wild‐type plumage coloration, this brown female successfully raised all four chicks, all of which grew wild‐type feathers. Having access to the full social family presented a unique opportunity to examine how, despite a plumage coloration that would normally be selected against, a bird could successfully find a mate and rear nestlings. Our objectives were to determine genetic relationships in this social family and explore possible circumstances for the female's apparent successful mating.

## 2 MATERIALS AND METHODS

### 2.1 Study species and site

A year‐round resident population of Eastern Bluebirds in northeast Arkansas (35°54′ N; 90°40′ W) has been monitored since the 2003 nesting season (Harrod & Rolland, 2020). The study site has about 150 accessible nest boxes. Other nest boxes, inaccessible to us, are available to bluebirds in the study area. The focal nest box, Box 601A (35°54′54.56″ N; 90°39′52.19″ W), is the easternmost point of our study site and is 800 m away from the nearest monitored nest box.

In that population, the nesting season runs from mid‐March to late August. Eastern Bluebirds are socially monogamous, but multiple parentage, mostly through extra‐pair paternity, within a brood is common (Gowaty & Karlin, 1984; Gowaty & Plissner, 2020). Although only the female builds the nest and incubates the eggs, both parents provide parental care. Within a breeding season, they typically attempt to raise up to three broods, although a fourth brood is possible (Harrod et al., 2021). Each brood comprises up to six chicks.

### 2.2 Field observations and methods

As part of the long‐term bluebird monitoring program, we monitored each nest box every 1–7 days depending on its status (i.e., no nest, nest in construction, incubation, and chick rearing). We found a nest with three bluebird eggs in the focal nest box on July 18, 2022. On July 20, 2022, we saw the female for the first time, flying out of the box. Our initial impression was of a pale bird, possibly a different species. Using binoculars, we confirmed she was an adult bluebird, unbanded. We found a fourth egg in the nest that same day. We did not find more eggs on July 22, 2022; eggs were being incubated. The first egg hatched on August 1, 2022. The following day, two more eggs hatched, and by August 3, 2022, all eggs had hatched.

Following our monitoring program protocol, we banded and measured both female and male social parents following hatching, that is, on 2 and August 3, 2022, respectively, using a trap designed by Robinson et al. (2004). Parents were aged based on the relative amount of white on the edges of the tenth primary covert (Pyle, 1997). We also banded and measured all chicks on day 13, that is, on August 13, 2022. We measured body mass to the nearest 0.5 g using a Pesola scale, relaxed wing chord, and outer right rectrix length to the nearest 1 mm using an elbowed ruler. Chicks were sexed based on wing color (Gowaty & Plissner, 2020). Adults were given a numbered USGS aluminum band and three colored bands in a unique combination, whereas chicks received only one color band on one leg and the USGS band on the other leg. We then checked the nest daily starting August 15, 2022 to monitor fledging. All chicks had fledged when we visited the nest early morning on August 20, 2022.

In addition to the regular monitoring protocol, for this study, we also took blood samples from both adult birds and all four chicks to establish relatedness. Using a 27 G sterile BD PrecisionGlide needle to puncture the brachial vein (Owen, 2011), we collected about 100 μL of blood (i.e., <1% of the bird body mass) from each bird with heparinized capillary tubes (ThermoFisher Scientific, Waltham, Massachusetts). We conducted handling and sampling protocols as approved by the Arkansas State University Institutional Animal Care and Use Committee (#FY20‐21‐268) and under the Arkansas State Scientific Collection Permit #122120211 and US Geological Survey banding permit #23811.

### 2.3 Genetic analysis

We extracted genomic DNA from each of the six blood samples using a DNeasy Blood and Tissue Kit (Qiagen, Hilden, Germany) and assessed DNA quantity using a DeNovix fluorometric assay (DeNovix, Wilmington, Delaware). All samples measured above the 10 ng/μL target (26.5–143.9 ng/μL) and were shipped to Biomarker Technologies Corporation (Hong Kong, China) for library preparation and whole‐genome sequencing. Indexed samples were pooled and sequenced on an Illumina NovaSeq 6000 S4 (Illumina, California), generating 150 bp paired‐end reads with a target average coverage of 5× (based on a 1.1 Gbp genome size). We trimmed raw sequence reads using Trimmomatic v.0.39 (Bolger et al., 2014) by removing leading and trailing bases with PHRED scores <3, trimming 4 bp windows with average PHRED scores <15, and removing any trimmed reads <75 bp. We also removed duplicate reads using BBMap v.38.82 (Bushnell, 2014). We checked the quality of reads before and after trimming using FastQC v0.11.5 (Babraham Bioinformatics). We then mapped trimmed reads to a high‐coverage genome assembly of *S. sialis* (NCBI GenBank: GCA_009812075.1) using Bowtie2 v.2.4.1 (Langmead & Salzberg, 2012), including reads that were used to generate the reference assembly (GenBank SRA #SRR9891081). We jointly called single nucleotide polymorphisms (SNPs) in BCFtools v.1.10.2 (Danecek et al., 2021) and filtered resulting SNPs based on mapping quality (<20), base quality (<28), depth (<20 & >100), and missing data using BCFtools and VCFtools v.0.1.15 (Danecek et al., 2011).

We performed a paternity analysis on all six individuals by estimating the KING relatedness coefficient in Relatedness2 in VCFtools (Manichaikul et al., 2010). To test for maternal lineages among our samples, we assembled the mitogenomes from each sample using MITObim v.1.9.1 (Hahn et al., 2013) with a *nad2* sequence from NCBI GenBank (accession AY049510) as the starting seed. We then annotated the resulting contigs in MITOS (Bernt et al., 2013) and extracted the *nad2* gene for each sample using Geneious Prime v.2022.0.2 (Biomatters, Ltd.). We aligned *nad2* (including the reference sequence as an outgroup) using the MAFFT v.7.490 (Katoh & Standley, 2013) plugin in Geneious. We then calculated uncorrected distances (*p*‐distances) and generated a neighbor‐joining (NJ) tree with a K2P (“K80”) substitution model using the APE v.5.6‐2 package (Paradis & Schliep, 2019) in R v.4.0.2 (R Core Team, 2022).

## 3 RESULTS

**FIGURE 1:**
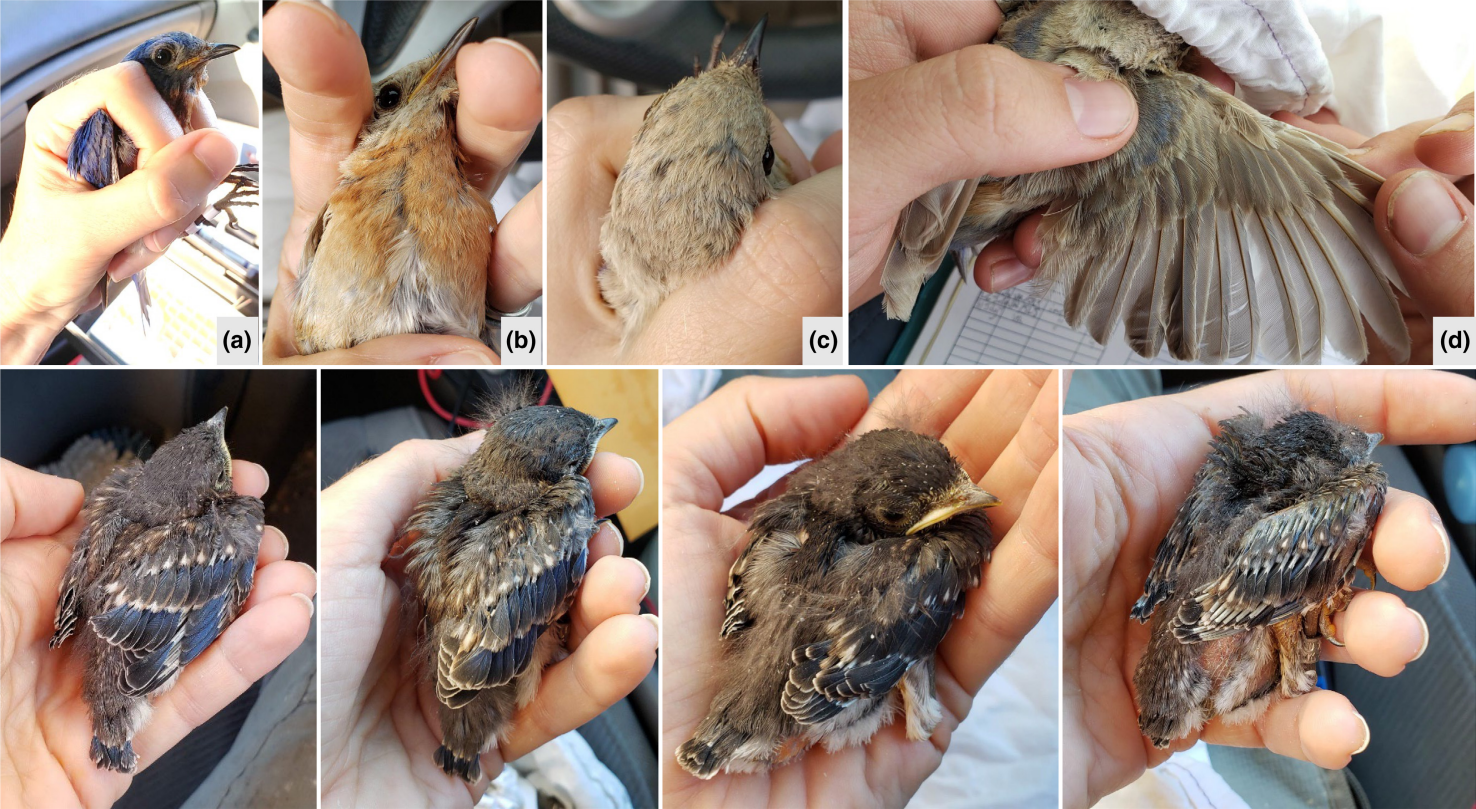
Social family of Eastern Bluebirds (*Sialia sialis*) in Jonesboro, Arkansas, in August 2022. The adult male presents typical plumage (a), whereas the adult female exhibits typical breast and eye coloration (b) but beige head (c), back, wings, and tail aside from a few blueish scapular feathers (d). Chicks grew typical plumage (bottom panel; from left to right: two males, one female, and one smaller chick of unknown sex).

Both adults presented morphological measurements within the normal range at our site in 2022 (Appendix 1: Table A1). We did not detect any abnormality (e.g., crossed bill, evidence of avian pox on legs or bill) in either parent (Figure 1). In terms of plumage, the female's breast was a normal chestnut and her eyes a normal black but the head, back, wings, and tail were a beige color except for a few blueish scapular feathers. The female could not be properly aged due to her discoloration. The wild‐type male was a second‐year bird. Chicks were also within the range of expected measurements (Appendix 1: Table A1). None of the chicks exhibited signs of aberrations in feather coloration or their skin coloration (Figure 1). The smallest chick (C_4_) was not developed enough for sexing.

**FIGURE 2:**
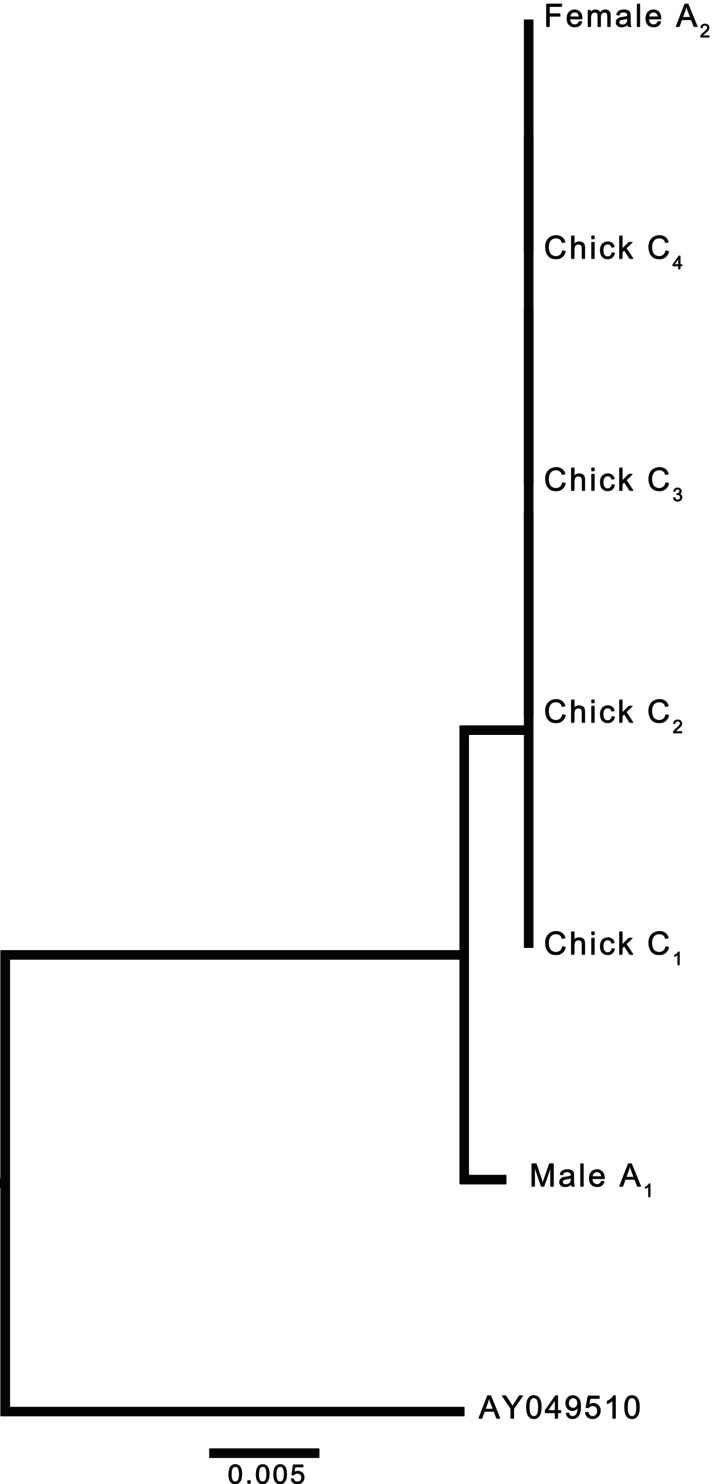
Neighbor‐joining distance tree of seven *Sialia sialis* individuals generated from mitochondrial *nad2* sequences. Female A_2_ and Male A_1_ were a social pair and Chick C_1_–C_4_ were the four chicks in the nest. The tree is rooted in a *S. sialis* sequence obtained from GenBank (accession AY049510).

We obtained an average of 58,451,744 raw reads, 39,515,989 post‐trimming reads, and 10,094,823 SNPs (Appendix 2: Table A2). The brown female was not related to her social mate and was only related to the second degree to her social offspring (Table 1), suggesting that she was either an aunt or grandmother. Similarly, the social father was only related to the second degree to the chicks (Table 1). Finally, three chicks were true siblings, but chick C_4_ had a lower relatedness value (Table 1), suggesting that it was a half‐sibling. The mitochondrial DNA further indicated that all chicks had the same maternal lineage, suggesting that the half‐sibling was sired by a different male. The female and all four chicks had *p*‐distances of 0.000, whereas the male had *p*‐distances of 0.005 with each chick and the female (Figure 2, Appendix 3: Table A3).

**TABLE 1:** Relatedness values of an Eastern Bluebird (*Sialia sialis*) social family in Jonesboro, AR, in August 2022.

	Social parents		Social offspring				Ref
	Male A_1_	Female A_2_	Chick C_1_	Chick C_2_	Chick C_3_	Chick C_4_	
Male A_1_	0.5						
Female A_2_	−0.099	0.5					
Chick C_1_	0.154	0.158	0.5				
Chick C_2_	0.155	0.161	0.220	0.5			
Chick C_3_	0.160	0.166	0.203	0.173*	0.5		
Chick C_4_	0.143	0.147	0.139	0.170	0.105	0.5	
Ref	−0.076	−0.072	−0.083	−0.080	−0.073	−0.093	0.5

*Note*: The reference individual (Ref) is from the NCBI GenBank (GCA_009812075.1). Relatedness values are 0.5 for self (salmon shade), 0.177–0.354 for a first‐degree relationship (i.e., full sibling, parent‐offspring; peach shade), 0.088–0.176 for a second‐degree relationship (i.e., grandparent‐grandchild, aunt/uncle‐niece/nephew, or half‐sibling; green shade), and <0.044 for unrelated individuals (blue shade; Manichaikul et al., 2010).

^*^ This value should be interpreted as a second‐degree relationship. However, because the pairs C_1_–C_2_ and C_1_–C_3_ are first‐degree relationships, C_2_ and C_3_ must be related to the first degree. Female A_2_ has a brown phenotype.

## 4 DISCUSSION

**FIGURE 3:**
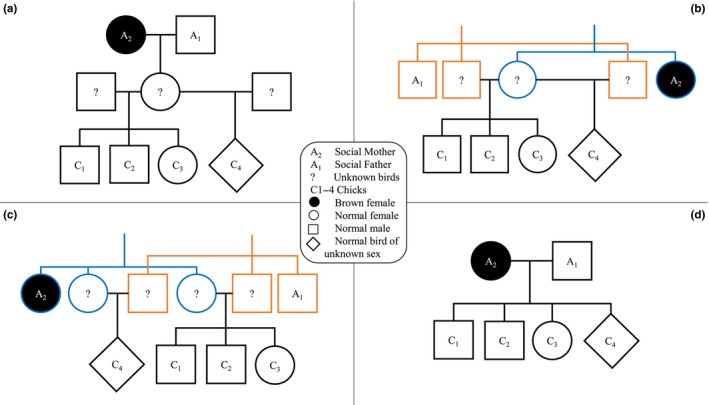
Possible genetic relationships between a brown female Eastern Bluebird (*Sialia sialis*), her social mate, and apparent offspring in August 2022, in Jonesboro, Arkansas. Lines of different colors (orange vs. blue) highlight different ancestries. The brown female may have been a grandmother if the social father was aged incorrectly in the field (a). The brown female may also have been a second‐year aunt that raised the offspring of either one sister who mated with two brothers (b) or two sisters that mated with brothers (c). In a fourth scenario where all our relatedness values are erroneous, the brown female could have been the direct mother of all four chicks (d).

The brown phenotype is most commonly expressed in females (van Grouw, 2021). In July 2022, in Jonesboro, AR, we encountered an Eastern Bluebird female with a brown phenotype. Despite a seemingly unattractive plumage, this brown female had paired with an unrelated mate and raised four offspring. The social parents were both only related to the second degree to the offspring which all shared the same maternal lineage. One offspring was a half‐sibling, implying extra‐pair paternity. However, extra‐pair paternity is frequent in Eastern Bluebirds, with 8%–20% of nestlings or 9%–27% of broods being from extra‐pair sires (Gowaty & Karlin, 1984; Meek et al., 1994; Stewart et al., 2010). We propose four family trees from most to least to probable (Figure 3) and discuss the implications of this unusual pairing within each scenario.

In the first scenario, the brown female and her mate would be grandparents to their social offspring (Figure 3a). Together, they would have raised a daughter in 2021 that mated in 2022 with two unrelated males (Figure 3a). We do not know the year of birth of this brown female. If she were a grandmother, she was at least ~2 years old (born in 2020) but may have been older. The oldest records from banding data indicate a maximum lifespan of 6–7 years (Gowaty & Plissner, 2020). By contrast, the social father would have been incorrectly aged as a second‐year instead of an after‐second‐year male. Pyle (1997) recommends aging Eastern Bluebirds using the relative amount of white on the tip of the tenth primary covert. However, based on recaptures of birds banded as chicks over the past 12 years, we found that this method is not always reliable (unpublished data). Pyle handled this species infrequently (personal communication, 2022), and in the newest version of the Identification Guide to North American Birds (Pyle, 2023), he suggested it be further studied. A pair of two after‐second‐year birds is consistent with Siefferman and Hill's (2005) findings that Eastern Bluebirds tend to mate assortatively by age.

The genetic mother (brown female's daughter in this scenario) did not incubate her own eggs and the brown female must have taken over the nest as a result of either nest usurpation or kin selection. Nest usurpation is common among cavity‐nesting birds (Lindell, 1996), although cases of conspecific nest usurpation seem rare (Berl et al., 2013; Gonzáles‐Crespo & Puente‐Rolón, 2021; Leffelaar & Robertson, 1985). Eastern Bluebirds are fierce nest usurpers of other species (Gowaty & Plissner, 2020; Rowe & Phillips, 2016), but conspecific nest usurpation has never been reported (Gowaty & Plissner, 2020). In addition, for our focal nest, the putative usurper did not lay eggs of her own, likely because the new female, as the genetic mother's mother, already shared genetic material with the existing eggs. Still, such intrafamily nest usurpation is surprising. Finally, nest usurpation usually occurs when nest sites are limited (Lindell, 1996), but that was not the case in our study site because the February 2021 snowstorms caused an 87% decline in the population (unpublished data).

If kin selection occurred, the genetic mother most likely died soon after laying her fourth egg and her mother (the brown female) took over incubation. Alternatively, perhaps the genetic mother attempted to maximize her fitness by starting another nest elsewhere. In both alternatives, the brown female increased her fitness by providing parental care to the nest of a direct relative (daughter in scenario 1). However, Eastern Bluebirds rarely engage in kin selection if at all. Only 0.1% of nesting attempts in South Carolina were the results of two females laying eggs in the same nest and sharing parental care; interestingly, two of these nesting attempts involved a mother‐daughter partnership (Gowaty & Plissner, 2020). By contrast, cooperative breeding is common for Western Bluebirds (*S. mexicana*), although help is never from females and never during incubation (Dickinson et al., 1996).

Although cooperative breeding is rare in Eastern Bluebirds, offspring born late in the season tend to stay with parents over the winter and in the natal area the following breeding season (Gowaty & Plissner, 2020; Lang, 2013). The genetic mother's parents (the brown female and her mate) were not captured in 2021 but may have nested in a nearby natural cavity or an unknown nest box; the focal box was repeatedly occupied by House Sparrows (*Passer domesticus*) until June 19, 2021 and again until May 30, 2022. Furthermore, natal dispersal is typically biased toward females in Eastern Bluebirds (Plissner & Gowaty, 1996) but not always (Lang, 2013), and late‐born offspring in better body condition are more likely to stay (Lang, 2013). At our site, 27% of the females recaptured as breeders nested within 200 m of the natal box (unpublished). Therefore, we speculate that the genetic mother was a late‐born offspring in good body condition (from low competition since the 2021 snowstorm) who stayed in the natal area.

In the second scenario, the brown female would be a second‐year aunt that raised the offspring of one sister (Figure 3b). That sister would have mated with two brothers, one of which sired chicks C_1_–C_3_ and the other sired C_4_. This scenario would imply that the social father was a brother to the two genetic fathers and, therefore, an uncle to all chicks. Finally, two sisters from one family and three brothers from another family would have had to survive their first year and stayed in or dispersed to the same area. Although juveniles form multifamily groups that can fly kilometers at a time (Gowaty & Plissner, 2020), it seems unlikely that two fraternal dispersal events occurred in the same area. At our study site, of 325 birds banded as chicks in 2012–2021 and recaptured as breeders, no instance of two same‐sex sibling groups were recaptured in the same breeding area (unpublished data). Furthermore, like the first scenario, this second scenario would involve intrafamily nest usurpation.

In the third scenario, the brown female (as a second‐year aunt) would have raised the offspring of her two sisters (Figure 3c). One sister “a” would have laid the first three eggs and a second sister “b” would have laid the fourth egg. The sisters would have mated with brothers. Consequently, the fourth offspring, instead of being a half‐sibling, would have been a super first cousin sharing the same paternal and maternal grandparents. Like in the second scenario, the social father would be the brother of the two genetic fathers. This scenario seems less probable than the other two scenarios because, in addition to the dispersal requirement described for the second scenario, brood parasitism between sisters followed by nest usurpation by a third sister would be necessary. In general, conspecific brood parasitism is mostly anecdotal in Eastern Bluebirds, with <1% of nestlings as the product of egg dumping (Gowaty & Plissner, 2020). Because of the snowstorm‐caused mass mortality, nest sites were not a limiting factor triggering conspecific brood parasitism. However, sister “b” could have had her nest destroyed and dumped her last egg in the nest of sister “a,” which may have died before the brown sister took over. The number of unlikely events required for this scenario makes it least probable.

Other pair combinations were possible with, for example, the brown female as a grandmother and her mate as an uncle to the social offspring, or vice versa. However, we assumed assortative mating by age (Siefferman & Hill, 2005) in the second and third scenarios to minimize the number of conditions (e.g., fraternal dispersal, conspecific brood parasitism). With this assumption, the brown female as a second‐year bird would have been born in 2021, just after the population suffered the mass mortality event caused by the snowstorm. A population bottleneck can be associated with an increased probability of inbreeding and reduced genetic diversity in the short term (Nei et al., 1975). Bottlenecks are also more likely to remove rare alleles from a population but can increase the frequency of deleterious recessive alleles in subsequent generations (Kirkpatrick & Jarne, 2000; Luikart et al., 1998). The effects of a genetic bottleneck can occur more rapidly for Z‐linked loci (Pool & Nielsen, 2007; Schield et al., 2021), although we do not have direct evidence that the Brown allele increased in bluebirds as a result of the bottleneck.

The last scenario reflects a traditional model where social relationships match genetic relationships, i.e., the brown female and her mate are the genetic parents of all offspring (Figure 3d). Although the most parsimonious, this scenario would imply that our relatedness values are erroneous. Our sample was small, which could have led to less reliable estimates of relatedness (Wang, 2017). However, we ran our analyses with and without a completely unrelated bluebird reference sequence and obtained similar results, suggesting that our relatedness values are correct.

Regardless of the scenario, the brown female was able to find a mate and successfully fledged a brood of four chicks, even though typically females with more‐ornamented structural plumage are associated with higher reproductive success (Siefferman & Hill, 2005). If the brown female and her mate were grandparents, it means that they mated in 2021, when mating options were limited because of the preceding snowstorm, and then stayed together in 2022. Pairs that are successful are more likely to stay together (Pinkowski, 1977), and this pair, if grandparents, had been successful in 2021, given that their daughter produced the clutch/brood they raised in 2022. By contrast, if the brown female's mate was a second‐year uncle (second and third scenarios), his benefit in partnering with a discolored female is unclear because he would have had more potential partners in 2022.

## AUTHOR CONTRIBUTIONS

**Joseph L. Schroeder:** Conceptualization (supporting); funding acquisition (lead); methodology (supporting); writing – original draft (lead); writing – review and editing (supporting). **Alexander J. Worm:** Data curation (lead); formal analysis (lead); methodology (lead); writing – original draft (equal); writing – review and editing (supporting). **Andrew D. Sweet:** Conceptualization (equal); methodology (supporting); supervision (equal); validation (lead); visualization (equal); writing – review and editing (equal). **Virginie Rolland:** Conceptualization (lead); methodology (equal); project administration (lead); supervision (equal); writing – original draft (equal); writing – review and editing (lead).

## CONFLICT OF INTEREST STATEMENT

Authors have no conflict of interest to declare.

## Data Availability

Raw genomic sequence data will be available on the NCBI SRA database (accession pending). Relevant data files are available on Dryad at https://datadryad.org/stash/share/KI5Pv813idX57cgUuDqGVVa6ijR38WKBI_IB7zhD2Y0. The associated DOI will be 10.5061/dryad.ht76hdrnn.

## APPENDIX 1

**TABLE A1:** Measurements for two adult Eastern Bluebirds (*Sialia sialis*) and their four social offspring in August 2022, in Jonesboro, Arkansas.

ID	Sex	Wing chord (mm)	Tail length (mm)	Body mass (g)
A_1_	M	99	63	29
Average adult male		99.7 (0.6; 95–106)	63 (0.5; 60–68)	29.3 (0.3; 26.5–32.5)
A_2_	F	94	57	28.5
Average adult female		94.9 (0.4; 89–98)	58.4 (0.5; 53–63)	29.9 (0.4; 24.5–35)
C_1_	M	52	15	28
C_2_	M	52	15	30
C_3_	F	50	14	28
C_4_	U	45	9	26
Average Day13 chick		51 (0.2; 38–59)	14 (0.2; 6–19)	27.5 (0.2; 20–33)

*Note*: For comparison, the average (SE; range) within the population in 2022 is provided for each adult sex (*n*_male_ = 23, *n*_female_ = 28) and 13‐day‐old chicks (*n* = 246). When sexing as male (M) or female (F) was not possible, we recorded the bird as unknown (U). Tail length is the length of the sixth right rectrix.

## APPENDIX 2

**TABLE A2:** Number of raw and post‐trimming reads used to determine relatedness in a *Sialia sialis* social family in summer 2022, in Jonesboro, Arkansas.

Sample name	Number of raw reads	Number of reads post‐trimming
Male A_1_	58,543,628	37,320,723
Female A_2_	54,540,538	39,402,305
Chick C_1_	55,832,944	37,428,481
Chick C_2_	55,275,654	37,945,001
Chick C_3_	58,252,708	40,392,307
Chick C_4_	47,276,268	32,521,981
Reference	79,440,468	51,601,130

*Note*: Genomic data were included from an unrelated individual as a comparison (NCBI SRA accession SRR9891081). Total number of single nucleotide polymorphisms used was 10,094,823.

## APPENDIX 3

**TABLE A3:** Uncorrected pairwise distances (*p*‐distances) for seven *Sialia sialis* individuals calculated from mitochondrial *nad2* sequences.

	Outgroup	Male A_1_	Female A_2_	Chick C_1_	Chick C_2_	Chick C_3_	Chick C_4_
Outgroup	0						
Male A_1_	0.042512	0					
Female A_2_	0.043478	0.004831	0				
Chick C_1_	0.043478	0.004831	0	0			
Chick C_2_	0.043478	0.004831	0	0	0		
Chick C_3_	0.043478	0.004831	0	0	0	0	
Chick C_4_	0.043478	0.004831	0	0	0	0	0

*Note*: Male A_1_ and female A_2_ were a social pair, and chicks C_1_ through C_4_ were the four chicks in the nest. The seventh sequence is from an unrelated individual (NCBI GenBank accession AY049510).
